# Noninvasive Prediction of Language Lateralization Through Arcuate Fasciculus Tractography in Patients With Low-Grade Gliomas: Correlation With The Wada Test

**DOI:** 10.3389/fonc.2022.936228

**Published:** 2022-07-22

**Authors:** Dongdong Wu, Meng Zhang, Jiefeng Geng, Xiaolei Chen

**Affiliations:** ^1^ Department of Neurosurgery, Chinese People's Liberation Army (PLA) General Hospital, Beijing, China; ^2^ Department of Neurosurgery, The Second Hospital of Southern Theater of Chinese Navy, Sanya, China; ^3^ Department of Neurosurgery, First Affiliated Hospital of Zhengzhou University, Zhengzhou, China

**Keywords:** arcuate fasciculus, DTI, fractional anisotropy, glioma, lateralization, LGG, tractography, Wada

## Abstract

Language lateralization is unique to humans, so clarifying dominant side is helpful for removing gliomas involving language areas. This study investigated the arcuate fasciculus (AF) reconstructed by diffusion tensor imaging–based tractography (DTT) in predicting language lateralization in patients with low-grade gliomas. Wada test was performed to determine the language Dominant Hemisphere (DH) and the Contralateral Hemisphere. DTI data [1.5-T magnetic resonance imaging (MRI)] was used to reconstruct AF by two independent operators using a DTT method. Fiber number, volume, and fractional anisotropy (FA) of bilateral reconstructed AF were measured. Lateralization indexes (LIs), including Number Index (NI), Volume Index (VI), and FA Index (FI), were accordingly calculated by mean values. A total of 21 patients with WHO Grade II gliomas in the left hemisphere were included. Every patient received a successful Wada test and reconstruction of bilateral AF. DTT metrics of reconstructed AF, such as fiber number, volume, and FA, showed significantly asymmetric between hemispheres. All the LI (NI, VI, and FI) values were statistically higher in the DH determined by the Wada test. No discrepancy was found between the prediction using the cutoff values of DTT metrics and the results of WADA test. The Kappa values were 0.829, 0.696, and 0.611, indicating NI and VI as more reliable predictor than FI although FI itself may also be feasible. Compared with the Wada test, we consider that DTT of AF is a non-invasive, simple, relatively accurate, and feasible method in predicting language lateralization in patients with low-grade gliomas.

## Introduction

Hemispheric language dominance is a defining characteristic of the human brain. For example, language function is lateralized to the left hemisphere in 75%~98% of the healthy right-handed volunteers and 53%~81% of the left-handed volunteers (1).

Arcuate fasciculus (AF), a type of crucial fibers in human language system, is proposed as a classic model for language production and comprehension and links the territory of Broca and Wernicke (2). Moreover, diffusion tensor imaging (DTI)–based tractography (DTT) is a non-invasive technique and has been widely reported for the reconstruction of white matter (WM) fibers including AF *in vivo* (3). DTT makes visualization of the AF possible, and the feasibility and accuracy have been confirmed (4–8). A few studies have so far focused on presurgical planning, DTT for AF, and postoperative outcome (9). Moreover, the correlation between asymmetry of reconstructed AF and language lateralization has been further investigated (10–12).

Noteworthily, the boundary of LGG is not clear compared with high-grade glioma, and postoperative neurological dysfunction is more likely to occur (13, 14), so non-invasive preoperative investigation of the relationship between dominant AF and LGG’s location would reduce the probability of postoperative language dysfunction. The Wada test is known as such a gold standard for preoperative assessment of language lateralization (15). However, it is invasive, costly, and unpleasant experience for patients and could even result in complications or adverse effects (16, 17).

In recent years, researchers have been devoted to non-invasively distinguishing dominant hemispheres. Although they found the concordance between DTI and Wada test in predicting language lateralization for patients with epilepsy (18–21), for patients with LGG, this concordance has not yet been well addressed. Therefore, on the basis of our previous studies of AF (22, 23), this study focused on LGG and is to investigate the feasibility of the asymmetry of AF reconstructed by DTT in predicting language lateralization when compared with the Wada test.

## Materials and Methods

### Study Design and Ethics Statement

From August 2012 to October 2019, patients with low-grade glioma (WHO Grade II) in the left hemisphere were enrolled in the study. Baseline data were obtained from our electronic medical record system. Handedness was assessed by the Edinburgh Handedness Inventory (24). The Wada test was used to investigate language dominance. The inclusion criteria were as follows: 1. adult patients aged from 18 to 65 years old; 2. the tumor close to presumed language areas or fiber bundles; and 3. intact language function proved by the Western Aphasia Battery (WAB) test (25). The exclusion criteria were as follows: 1. patients with MRI imaging reconstruction showing that arcuate fasciculus was destroyed by a glioma; and 2. patients who were unwilling or unable to cooperatively receive the WADA test. This study has been approved by the Institutional Review Board of Chinese PLA General Hospital in accordance with the Declaration of Helsinki. All participants provided their informed consent to participate in this study.

### Wada Test Protocol and Procedure

Middle cerebral artery (MCA) propofol Wada test protocol (26) and methylprednisolone injection prior to propofol (27) were adopted and performed by a veteran interventional neurosurgeon using microcatheter (Renegade HI FLO, Boston Scientific, Natick, Massachusetts, 01760-1537, USA). Vital signs were monitored non-invasively throughout the procedure. The detailed procedure was as follow. The ending point of microcatheter was placed at M1 segment of one side MCA after the distribution of intracarotid cerebral artery (ICA), and MCA was studied by cerebral angiography. Patients were instructed to raise and maintain their contralateral upper limb and to keep counting up aloud. The propofol dilute solution (propofol: 5%; glucose solution = 10 mg: 10 ml) was soon injected slowly through the catheter until effective events (contralateral limbs hemiplegia and interruption of counting numbers) were observed. Hand strength, sensitivity, and language function were evaluated throughout the test. Additional solution could be injected with the maximum dose of 15 ml in one side. The other side of the hemispheres was evaluated about 30 min after the procedure of one side. The aphasia side was defined as the Dominant Hemisphere (DH), whereas the other side was defined as the Contralateral Hemisphere (CH).

### MRI Data Acquisition

MRI scans were performed with a 1.5-T scanner (Siemens Espree, Erlangen, Germany) and a standard head coil with four channels. The sequences and parameters of each sequence are listed in **Table 1**. T1 weighted imaging was acquired by the magnetization-prepared rapid-acquisition gradient-echo sequence, and the sequence was performed twice before and after intravenous administration of 0.2 ml/kg body weight of gadopentetate dimeglumine (Gd-DTPA) (Magnevist; Schering, Berlin, Germany). T1 weighted imaging with no Gd-DTPA was defined as 3D T1, and T1 weighted imaging with Gd-DTPA was defined as 3D T1+C. DTI was obtained by means of a single-shot spin-echo diffusion weighted echo planar imaging sequence in the transversal plane, and motion-probing gradients were applied along 20 directions with a high b-value of 1,000 s/mm^2^ after one null image (b = 0 s/mm^2^) in each acquisition.

**Table 1:** MRI sequences and parameters.

Sequences	TR(ms)	TE(ms)	Slice Thickness(mm)	SliceNumber	Voxel size(mm × mm × mm)	Matrix Size	FOV(mm × mm)	Repetition	Scan Time (min:s)
T1	1,650	3.02	1	179	1.0×1.0×1.0	256×256	250×250	1	5:18
T2	6,000	93	5	23	0.4×0.6×5.0	256×160	230×230	1	4:20
FLAIR	9,000	86	5	23	0.9×0.9×5.0	256×160	230×230	1	4:32
DTI	9,400	147	2.7	40	2.0×2.0×2.7	128×128	251×251	4	6:52

DTI, diffusion tensor imaging; FLAIR, fluid-attenuated inversion recovery; FOV, field of view; TE, echo time; TR, repetition time.

### Data Processing and AF Tractography

All of the DICOM data of abovementioned sequences were imported and processed in the software of iPlan 3.0.5 (BrainLAB, Feldkirchen, Germany) with “Eddy current correct”, “Smooth”, and “Motion correct” ticked for DTI data. All the sequences were merged with the basic dataset, 3D T1, by “Image fusion” module. AF tractography was performed using tensor deflection algorithm and two-Region of Interest (ROI) method, as described by Catani and his colleagues (28). Fractional anisotropy (FA) threshold of 0.20 and fiber length of 50 mm were used for AF tracking in this study. Given the study by Fernandez-Miranda et al. (29), only the parts of AF were reconstructed, such as those connecting posterior part of median and inferior frontal gyri, lower part of precentral gyrus (beyond classic Broca’s area), and posterior part of superior, median, and inferior temporal gyri (beyond classic Wernicke’s area), so other “noise” part was excluded. In addition, the object creations of tumor and cerebrum were based on T2 fluid-attenuated inversion recovery (FLAIR) and 3D T1, respectively.

### Asymmetry Evaluation of Reconstructed AF

Each of the fiber number, volume, and FA of reconstructed AF was obtained by an average of data from two operators who received more than 1-year standard training on fiber reconstruction. Lateralization index (LI) was calculated as follows:


$$LI=\frac{X_{Left}-X_{Right}}{X_{Left}+X_{Right}}$$

In the above formula, X stands for the fiber number, volume, or FA of AF. Accordingly, LI is deduced, like the fiber Number Index (NI), the fiber Volume Index (VI), and FA Index (FI), to assess the lateralization between two hemispheres. Hence, a positive LI indicates that DH is probably on the left, whereas a negative LI indicates that DH is probably on the right.

### Statistical Analysis and Bias Control

SPSS Statistics 24.0 (IBM) was used and a P-value of < 0.05 was considered to be statistically significant difference. Wilcoxon test was used to compare the DTT metrics between DH and CH, and McNemar test was used to test the consistency between the prediction of DTT metrics and the results of the Wada test. To avoid bias, fiber tracking was independently performed by the two operators who were blind to the results of both fiber tracking and Wada test.

## Results

### Populations

Patients’ profiles are presented in **Table 2**, including age, sex, handedness, pathological diagnosis, Wada test, and DTT metrics of AF. A total of 21 patients were included in this study. The mean age was 38.4 years old (range from 23 to 57). The gender ratio was 10 (male) versus 11 (female). The pathological diagnosis included oligodendroglioma, astrocytoma. The entity of oligoastrocytomas diagnosed earlier was roughly classified as LGGs (uncertain subtype) because of insufficient biomarkers of examination panel at that time. Among them, three patients showed language dominance on the right hemisphere according to the results of the Wada test. Among two patients with left handedness, one had language dominance on the right hemisphere.

**Table 2:** General data of patients and DTT metrics of arcuate fasciculus.

Patient No.	Age	Gender	Handedness	Diagnosis	Wada Dominant	Fiber Number			Fiber Volume (mm^3^)			FA		
						Left	Right	NI	Left	Right	VI	Left	Right	FI
1	50	F	R	Frontal, A	R	446	2123	−0.65	6,772	11562	-0.26	0.31	0.38	−0.1
2	24	F	R	Frontal, LGG (uncertain)	L	4,405	1181	0.58	7,903	6137	0.13	0.41	0.4	0.01
3	49	M	R	Temporal, A	L	1,930	75	0.93	5,435	3298	0.24	0.4	0.38	0.03
4	34	F	R	Insular, A	L	2,722	1461	0.3	8,783	5793	0.21	0.4	0.43	−0.04
5	23	F	R	Temporal, O	L	4,713	2219	0.36	13,546	953	0.87	0.41	0.41	0
6	56	M	R	Temporal, LGG (uncertain)	L	3,097	1343	0.4	7,880	4963	0.23	0.43	0.4	0.04
7	41	M	L	Frontal, A	L	2,971	1577	0.31	9,812	7658	0.12	0.44	0.42	0.02
8	45	M	R	Frontal, O	R	5,599	8128	−0.18	12,878	15032	−0.08	0.36	0.35	0.01
9	42	F	R	Insular and temporal, A	L	4,205	508	0.78	11,242	5500	0.34	0.4	0.4	0
10	28	M	R	Temporal, A	L	4,942	2446	0.34	11,521	9867	0.08	0.4	0.4	0
11	44	M	R	Insular, O	L	3,832	1853	0.35	8,124	5983	0.15	0.47	0.47	0
12	33	F	R	Temporal, A	L	2,423	3988	−0.24	12,013	14045	−0.08	0.42	0.41	0.01
13	57	M	R	Frontal and insular, A	L	3,780	719	0.68	12,630	4803	0.45	0.37	0.35	0.03
14	46	M	R	Temporal, O	L	2,288	590	0.59	15,318	10212	0.2	0.42	0.4	0.02
15	29	F	R	Frontal, LGG (uncertain)	L	932	395	0.4	13,712	6373	0.37	0.39	0.37	0.03
16	33	F	R	Frontal, A	L	9,634	6190	0.22	18,158	20714	−0.07	0.48	0.44	0.04
17	44	M	R	Frontal, O	L	1,973	117	0.89	13,576	3939	0.55	0.41	0.4	0.01
18	43	F	R	Insular, A	L	3,054	769	0.6	9,671	11568	−0.09	0.4	0.39	0.01
19	26	F	L	Frontal, A	R	6,154	6359	−0.02	12,901	15231	−0.08	0.41	0.42	−0.01
20	34	M	R	Insular, O	L	2,978	2217	0.15	15,679	14899	0.03	0.4	0.39	0.01
21	25	F	R	Frontal, LGG (uncertain)	L	2,484	501	0.66	9,173	5156	0.28	0.42	0.38	0.05

FA, fractional anisotropy; DTT, diffusion tensor imaging based tractography; A, astrocytoma; O, oligodendroglioma; NI, fiber number index; VI, fiber volume index; FI, fractional anisotropy index.

### Wada Test Consequence

Every patient received a successful Wada test, and only two patients had additional propofol injection. No one experienced Grade 3 side effects according to the Mikuni’s side effect classification (17), and only one and two patients exhibited Grade 2 and 3 side effect, respectively. All transient hemipareses during the test were fully recovered within 5 min and speech function within 10 min. No permanent complications after the Wada test were observed in this study.

### DTT of AF and Dominance Prediction

**Figure 1:**
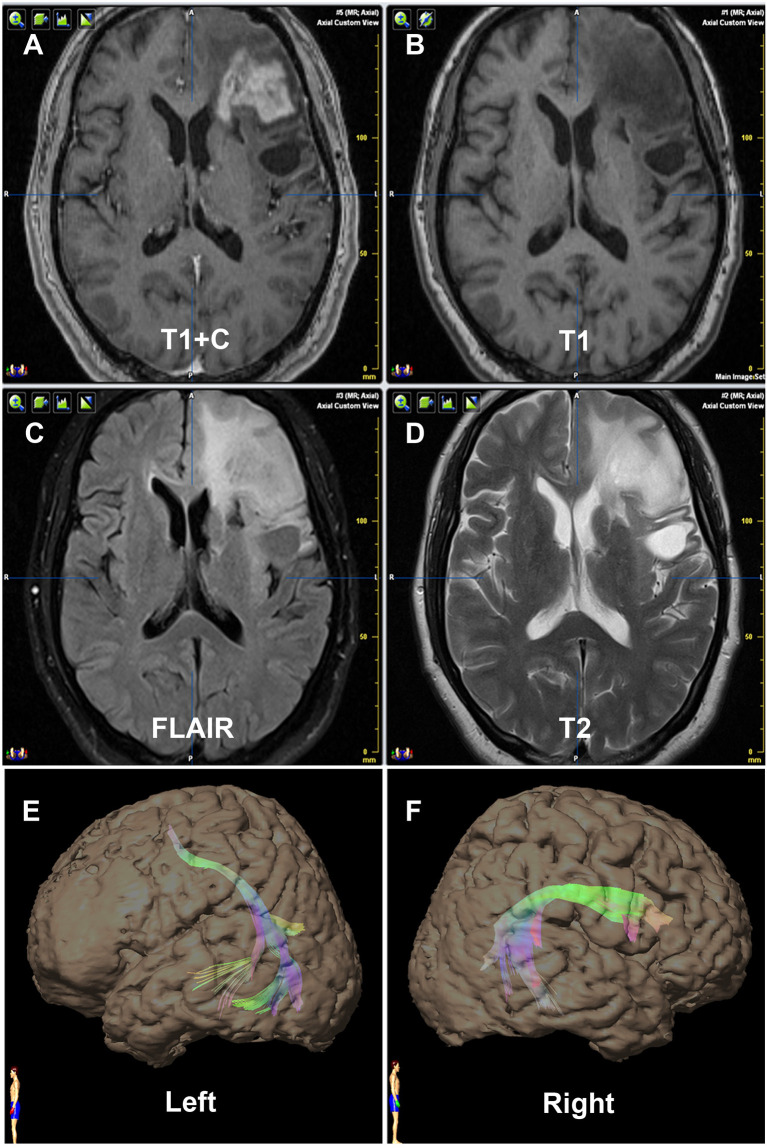
Contrast of bilateral AF by DTT of Patient No. 1. Panels **(A–D)** are preoperative MRI T1+C, T1, FLAIR, and T2, respectively, on one same transverse slice of Patient No. 1, who was diagnosed as recurrent astrocytoma in site in Broca’s area. Panels **(E, F)** show that the relative more fiber number and volume of AF are on the right hemisphere, which are in concordance with the language dominant side of the Wada test.

**Figure 2:**
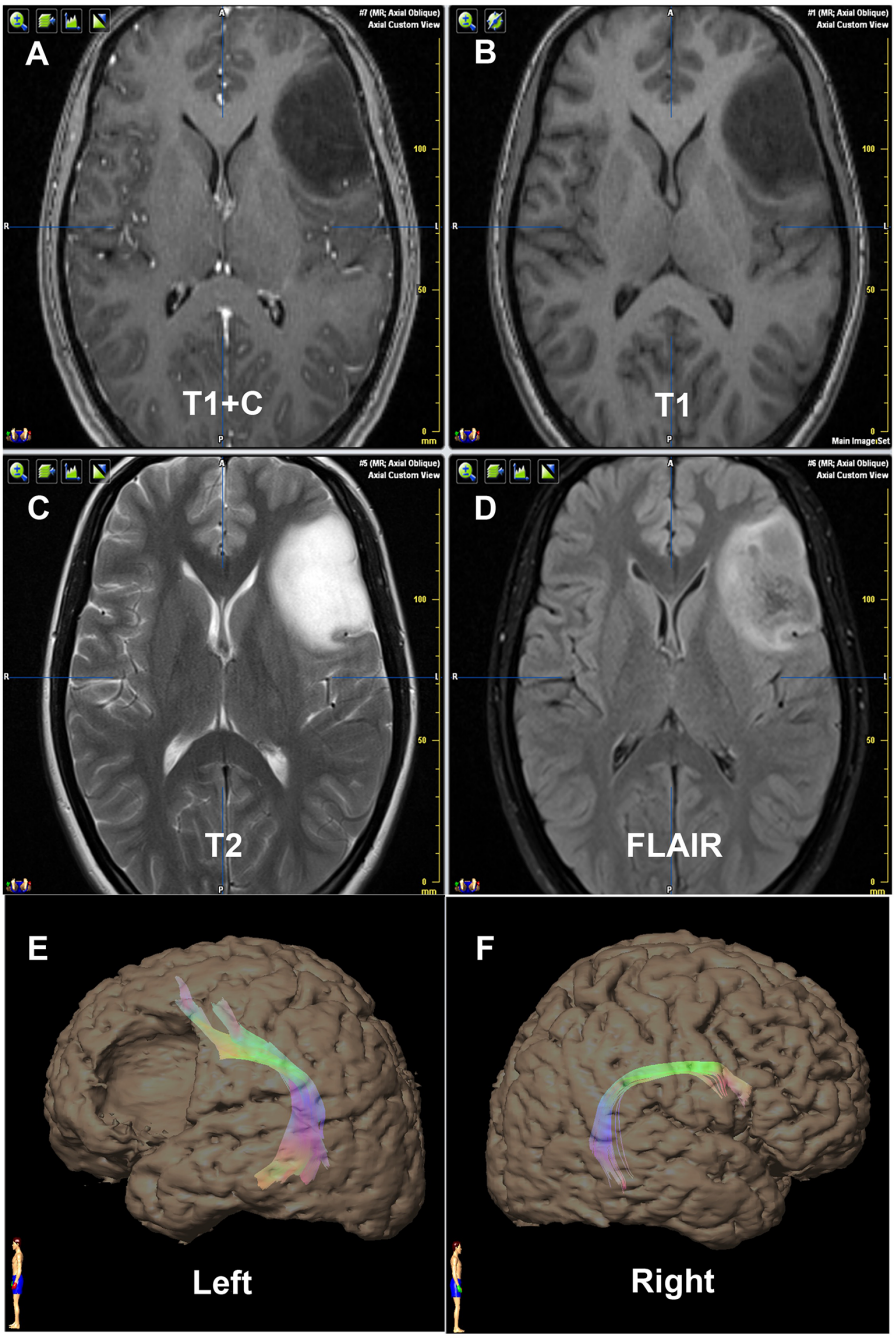
Contrast of bilateral AF by DTT of Patient No. 9. Panels **(A–D)** are preoperative MRI T1+C, T1, T2, and FLAIR, respectively, on one same transverse slice of Patient No. 9, who was diagnosed as primary astrocytoma in insular and temporal lobe. Panels **(E, F)** show that the relative more fiber number and volume of AF are on the left hemisphere, which are in concordance with the language dominant side of Wada test.

**Figure 3:**
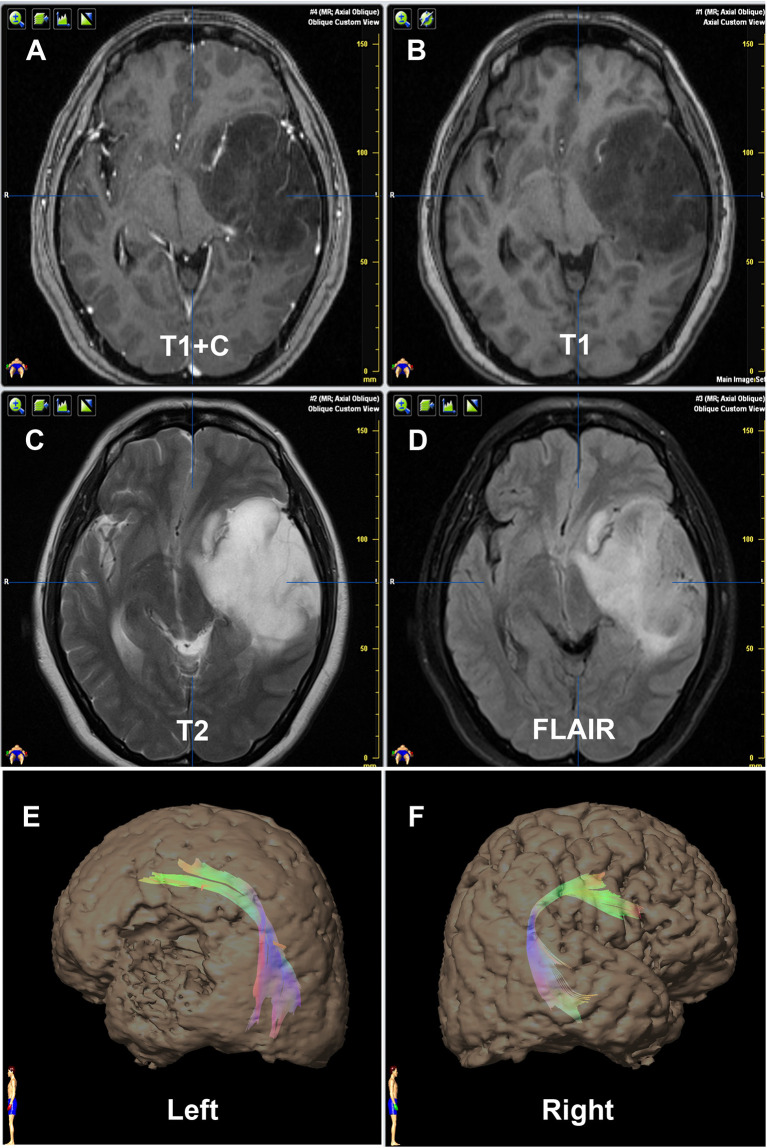
Contrast of bilateral AF by DTT of Patient No. 21. Panels **(A–D)** are preoperative MRI T1+C, T1, T2, and FLAIR, respectively, on one same transverse slice of Patient No. 21, who was diagnosed as primary LGG involving Broca’s area. Panels **(E, F)** show that the relative more fiber number and volume of AF are on the left hemisphere, which are in concordance with the language dominant side of Wada test.

**Figure 4:**
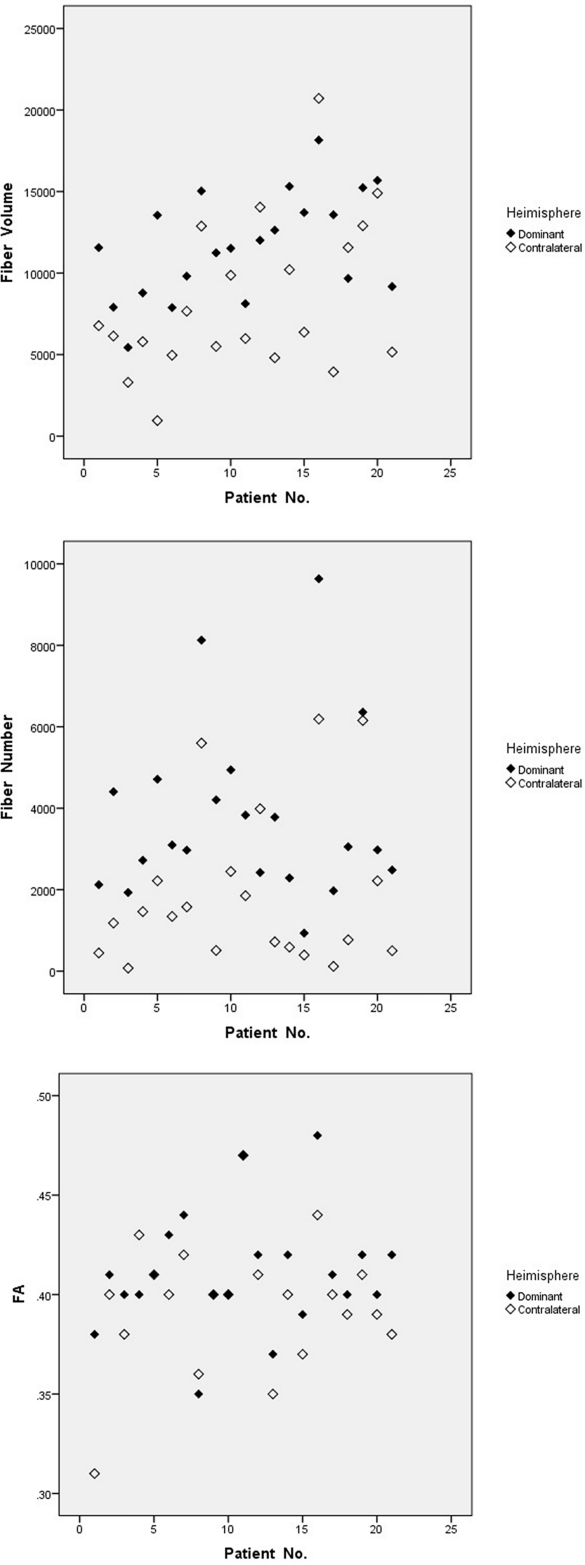
Discrepant metrics of DTT between dominant and contralateral hemisphere. Fiber number, fiber volume, and FA of AF show discrepant between DH and CH. FA, fractional anisotropy.

Bilateral AF of each patient was successfully reconstructed. Generally, each of DTT metrics of AF, such as fiber number, fiber volume, and FA, showed asymmetric between hemispheres (**Table 2**), similar to a contrast of bilateral AF in three illustrative cases (**Figures 1**–**3**). Moreover, all three patients with language dominance in the right hemisphere had lower or even negative LI values (NI, VI, and FI). We found a discrepancy of DTT metrics of AF between DH and CH (**Figure 4**). All the three metrics of LI (NI, VI, and FI) were statistically higher in the DH determined by the Wada test (**Table 2**), indicating LI values as potential predictor of DH.

**Figure 5:**
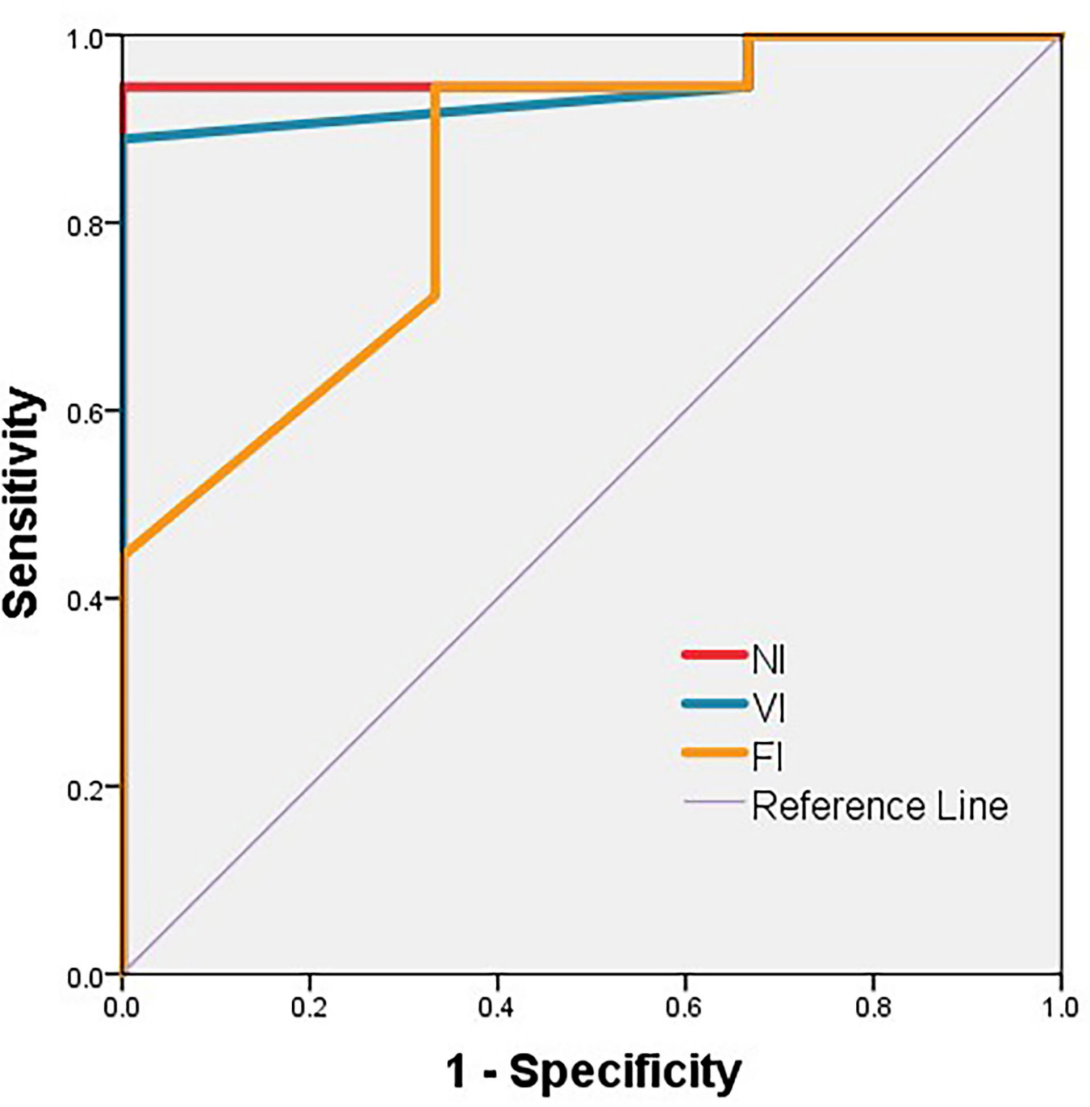
ROC curve of NI, VI, and FI. The area of ROC curve of NI, VI, and FI are 0.963, 0.944, and 0.843, respectively. The P-values are 0.012, 0.016, and 0.063, respectively. FI, fractional anisotropy index; NI, fiber number index; VI, fiber volume index.

As a result, NI, VI, and FI were taken into further analysis. On the basis of the respective cutoff values, these three metrics resulted in considerable sensitivities, specificities, and Youden Indexes for distinguishing DH (**Table 3**). The areas of ROC curve of NI and VI were 0.963 and 0.944 with significant P-values (0.012,0.016), although the area of FI was 0.843 with a borderline P-value (0.063) (**Figure 5**). Furthermore, no discrepancy was found between the prediction using the cutoff values of DTT metrics and the results of the Wada test (**Table 4**). The Kappa values of Fiber Number, Fiber Volume, and FA were 0.829, 0.696, and 0.611, respectively (**Table 5**). To summarize, language dominance prediction based on DTT metrics of AF, NI, and VI seems to be more reliable than FI although FI itself may also be feasible.

**Table 3:** Comparation of DTT metrics between dominant and contralateral hemisphere.

	Fiber Number Mean ± SD	Fiber Volume (mm^3^) Mean ± SD	FAMean ± SD
DH	3,760.6 ± 2,113.2	11,714.3 ± 3,240.5	0.4105 ± 0.0294
CH	1,921.3 ± 1,940.8	8,305.3 ± 4,751.2	0.3962 ± 0.0331
Z value ^a^	3.806	3.320	2.824
P-value (<0.05)	0.000^b^	0.001^b^	0.005^b^

DTT, diffusion tensor imaging based tractography; DH, dominant hemisphere; CH, contralateral hemisphere; FA, fractional anisotropy; SD, standard deviation.

a Wilcoxon test.

b Significant difference.

**Table 4:** Respective cutoff values of deduced DTT metrics and statistical analysis.

	NI	VI	FI
Cutoff	0.065	−0.075	−0.005
Sensitivity	0.944	0.889	0.944
Specificity	1	1	0.667
Youden Index	0.944	0.889	0.611

DTT, diffusion tensor imaging based tractography; FA, fractional anisotropy; FI, fractional anisotropy index; NI, fiber number index; VI, fiber volume index.

**Table 5:** The performance of DTT metrics to predict dominant hemisphere using defined cutoff values.

Wada Test	Fiber Number		Fiber Volume		FA	
	Left Dominate	Right Dominate	Left Dominate	Right Dominate	Left Dominate	Right Dominate
Left dominate	17	1	16	2	17	1
Right dominate	0	3	0	3	1	2
P-value (<0.05) ^a^	1.000		0.500		1.000	
Kappa value	0.829		0.696		0.611	

FA, fractional anisotropy.

a McNemar test.

## Discussion

Language lateralization is an interesting and enduring topic. In non-tumor patients with intracranial lesions, studies showed left dominance in 90% of the right-handed cases, 60% of the ambidextrous-handed, 43% of the left-handed (30), and 75.8% of the patients irrespective of handedness (15). For some patients with slow-growing tumors such as gliomas involving language areas, the dominant hemisphere was found to be likely located right originally (15, 30) or shift from left to right (31, 32). Jehna and colleagues also reported that patients with gliomas in the left hemisphere and intact language function tended to have an AF that was symmetric or lateralized to the right (33), indicating that contralateral AF may involve in patients harboring a glioma in the dominant hemisphere and may facilitate the recovery from aphasia and the reorganization of the language brain network even after surgical damage (9). A tumor actually contralateral to the dominant hemisphere would definitely make a neurosurgeon less constrained during a total or extended resection. Therefore, the exact information of language dominant hemisphere of each patient with intracranial tumor is helpful for making preoperative plan on balancing the reservation of language function and the extent of resection of a tumor involving critical normal functioning cortical areas and their WM connections (34, 35), not to mention an unclear and confusing boundary of low-grade gliomas under a microscope.

To avoid a preoperative invasive test, a number of non-invasive or minor invasive alternatives emerged for replacing the Wada test, including blood oxygen level–dependent functional MRI (BOLD-fMRI), functional transcranial Doppler, magnetoencephalography, near infrared spectroscopy, positron emission tomography, and single-photon emission computerized tomography (36, 37). A meta-analysis showed that the sensitivity and specificity of fMRI for atypical language dominance were 83.5% and 88.1%, respectively (38). The fMRI is safe, non-invasive, and widely used but also has its own limitations (15). There remains controversy regarding the correlation between the Wada test and fMRI although increased articles tried to establish their relationship (21, 39). Moreover, fMRI results are sometimes unreliable especially for intracranial tumors with some reasons (40–42). Even more, language tasks performance during a MRI scan may influence its results (43).

Compared with the Wada test and fMRI, DTT is obviously non-invasive and relatively simple because of a resting state during an MRI scan. DTI tractography is currently used for preoperative surgical planning to maximize the surgical resection avoiding to damage association and projection fibers located nearby the tumor (44, 45) and consistently reveals asymmetries of WM tracts between the two hemispheres (46, 47). The reconstruction of AF using DTT showed a significant leftward asymmetry in no less than 80% of healthy right-handers, with some being feasible only on the left side (47). Some researchers even consider AF as “non-resectable” tracts in contrast to other WM tracts that can be resected without inducing neuro-deficits (48). We are honorable to work on the reconstruction of the AF and previously reported a feasible method combining 1.5-T intraoperative MRI with AF neuronavigation to maximize resection and minimize language deficits when removing gliomas adjacent to AF (22), and we further found that the cutoff distance from a glioma to nearby AF was 3.2 mm for preventing aphasia, as seen on postoperative DTI-T (23). In fact, a number of studies demonstrated a correlation between DTI and fMRI, indicating that DTI could define language lateralization (12, 49, 50). Previous studies showed the concordances between DTT and Wada test ranged from 80% to 95.8% (18–21). However, in terms of LGG, there is a lack of studies analyzing the direct relationship between DTI and the Wada test.

The present study demonstrated that a language dominance prediction by the metrics of bilateral AF through DTT were consistent with a result of Wada test in patients with right-sided low-grade gliomas. Along with this study, Delgado-Fernandez et al. recently showed that fiber number, volume, and FA of AF were greater in the dominant hemisphere although they just focused on patients with epilepsy (18). Matsumoto et al. also found that the frontotemporal tract had more fibers and was greater in length, and the bundle was greater in volume in the dominant hemisphere (20). Similar to the effectiveness of VI and FI in this study, Tiwari et al. found a correlation between the result of the Wada test and fiber volume of the AF (P = 0.02) and a trend toward significance of FA values (P = 0.07) (21). Thus, it seems to be controversial to consider the laterality of FA of AF as a robust predictor of language lateralization in our patients with low-grade gliomas. However, higher FA values of the AF were actually found in the dominant hemisphere of the healthy volunteers (49, 50). Ellmore et al. also showed the same result in patients with epilepsy (19). We therefore consider that it is feasible to non-invasively predict language lateralization *via* the asymmetry of fiber number and fiber volume of AF in patients with low-grade gliomas although a prediction using FA needs further studies.

The surgical strategy is now dilemmatic for tumor resections nearby functional areas. Awake surgery is currently a standard method of functional areas detection, but not all patients are competent enough during awake surgery. Asleep surgery has less procedures, especially anesthesia difficulties. With this study, possibly boosting the prediction of WM tracts for asleep surgery, improving preoperative prediction accuracy, keeping patients motionless during an operation, and completing tumor resection while preserving neurological functions will be realized in the near future.

It is noted that Patient No. 12 was likely false positive in this study. However, this tumor was located in a temporal lobe, rather in a frontal lobe, when compared with other three right-dominant patients. Patient No. 12 did not have so many DTT fibers, and the asymmetry was not seemingly obvious either (**Table 2**). In fact, our data also showed that the tumor seemed to infiltrate the AF (not shown). All these factors may prevent our study from being perfect. With tumor site, AF infiltration, and reconstruction quality taken into accounts, more accurate results are worth waiting for.

Because a majority of patients have a dominant hemisphere on the left side, this study excluded tumors in the right hemisphere for a clinical purpose. Another limitation is relatively small sample size in total and a lack of patients with right-dominant hemisphere and those with left handedness, although we have had a largest number of participants with gliomas up to now. Furthermore, our system used only 1.5-T intraoperative MRI and DTI with 20 gradient directions. It is difficult to reconstruct some crossing and kissing fibers pertaining to distinct subcortical networks under such intensity of magnetic field (51). However, DTT has been improved by many researchers in recent years (52–54), and other fiber tractography techniques such as constrained spherical deconvolution tractography (55) and generalized q-sampling imaging–based tractography (29) are considered as promising methods to overcome the limitations of traditional DTT. Some researchers have started to investigate AF by new methods, and the results seem encouraging (29, 52–54). Moreover, as the researchers in different groups adopted different resolutions of enhancing the reliabilities of their results (19, 20) (21), we conducted a blind design and analyzed the mean value from two experienced operators, but the accuracy of DTT procedure depends on the operators because subjective biases could interfered to some extent (51). In addition to the AF, other WM tracts, such as the superior longitudinal fasciculus, inferior occipitofrontal fasciculus, uncinate fasciculus, and inferior longitudinal fasciculus, were also known to participate in language processing (7, 56, 57). They are definitely the directions of our research in the future.

## Conclusions

Sample size, imperfect tractography, and operator’s bias might constrain our research. However, in this study, DTT displayed as a non-invasive, simple, relatively accurate, feasible, and potential method in predicting language lateralization in patients with low-grade gliomas, especially when compared with the Wada test. Although the FA value of bilateral reconstructed AF needs to be further affirmed, we consider the fiber number and fiber volume are both reliable predictors of language lateralization, showing DTT as a promising method to compete with the Wada test in the future.

## Data Availability Statement

The raw data supporting the conclusions of this article will be made available by the authors, without undue reservation.

## Ethics Statement

The studies involving human participants were reviewed and approved by Ethics Committee of Chinese PLA General Hospital. The patients/participants provided their written informed consent to participate in this study.

## Author Contributions

Conceived and designed the study: XC. Performed the study: DW, MZ, and JG. Analyzed the data: JG. Wrote the paper: DW and MZ. All authors contributed to the article and approved the submitted version.

## Conflict of Interest

The authors declare that the research was conducted in the absence of any commercial or financial relationships that could be construed as a potential conflict of interest.

## Publisher’s Note

All claims expressed in this article are solely those of the authors and do not necessarily represent those of their affiliated organizations, or those of the publisher, the editors and the reviewers. Any product that may be evaluated in this article, or claim that may be made by its manufacturer, is not guaranteed or endorsed by the publisher.

## References

[1] Mazoyer B Laure Z Gaël J Crivello F Joliot M Perchey G. Gaussian Mixture Modeling of Hemispheric Lateralization for Language in a Large Sample of Healthy Individuals Balanced for Handedness. PloS One (2014) 9(6):e101165. doi: 10.1371/journal.pone.0101165 24977417 PMC4076312

[2] Geschwind N. The Organization of Language and the Brain. Science (1970) 170(3961):940–4. doi: 10.1126/science.170.3961.940 5475022

[3] Lerner A Mogensen MA Kim PE Shiroishi MS Hwang DH Law M. Clinical Applications of Diffusion Tensor Imaging. World Neurosurg (2014) 82(1-2):96–109. doi: 10.1016/j.wneu.2013.07.083 23916498

[4] Andrade CS Figueiredo KG Valeriano C Mendoza M Valente KD Otaduy MC. DTI-Based Tractography of the Arcuate Fasciculus in Patients With Polymicrogyria and Language Disorders. Eur J Radiol (2015) 84(11):2280–6. doi: 10.1016/j.ejrad.2015.07.014 26216794

[5] Bernard F Zemmoura I Ter Minassian A Lemee JM Menei P. Anatomical Variability of the Arcuate Fasciculus: A Systematical Review. Surg Radiol Anat (2019) 41(8):889–900. doi: 10.1007/s00276-019-02244-5 31028450

[6] Caverzasi E Hervey-Jumper SL Jordan KM Lobach IV Li J Panara V Racine CA. Identifying Preoperative Language Tracts and Predicting Postoperative Functional Recovery Using HARDI Q-Ball Fiber Tractography in Patients With Gliomas. J Neurosurg (2016) 125(1):33–45. doi: 10.3171/2015.6.JNS142203 26654181

[7] Incekara F Satoer D Visch-Brink E Vincent A Smits M. Changes in Language White Matter Tract Microarchitecture Associated With Cognitive Deficits in Patients With Presumed Low-Grade Glioma. J Neurosurg (2018) 130(5):1538–46. doi: 10.3171/2017.12.JNS171681 29882705

[8] Keser Z Sebastian R Hasan KM Hillis AE. Right Hemispheric Homologous Language Pathways Negatively Predicts Poststroke Naming Recovery. Stroke (2020) 51(3):1002–5. doi: 10.1161/STROKEAHA.119.028293 PMC7042036 31884909

[9] Di Cristofori A Basso G de Laurentis C Mauri I Sirtori MA Ferrarese C. Perspectives on (A)symmetry of Arcuate Fasciculus. A Short Review About Anatomy, Tractography and TMS for Arcuate Fasciculus Reconstruction in Planning Surgery for Gliomas in Language Areas. Front Neurol (2021) 12:639822. doi: 10.3389/fneur.2021.639822 33643213 PMC7902861

[10] Nucifora PG Verma R Melhem ER Gur RE Gur RC. Leftward Asymmetry in Relative Fiber Density of the Arcuate Fasciculus. Neuroreport (2005) 16(8):791–4. doi: 10.1097/00001756-200505310-00002 15891571

[11] Sreedharan RM Menon AC James JS Kesavadas C Thomas SV. Arcuate Fasciculus Laterality by Diffusion Tensor Imaging Correlates With Language Laterality by Functional MRI in Preadolescent Children. Neuroradiology (2015) 57(3):291–7. doi: 10.1007/s00234-014-1469-1 25467219

[12] Vernooij MW Smits M Wielopolski PA Houston GC Krestin GP van der Lugt A. Fiber Density Asymmetry of the Arcuate Fasciculus in Relation to Functional Hemispheric Language Lateralization in Both Right- and Left-Handed Healthy Subjects: A Combined fMRI and DTI Study. Neuroimage (2007) 35(3):1064–76. doi: 10.1016/j.neuroimage.2006.12.041 17320414

[13] Sun GC Chen XL Zhao Y Wang F Hou BK Wang YB. Intraoperative High-Field Magnetic Resonance Imaging Combined With Fiber Tract Neuronavigation-Guided Resection of Cerebral Lesions Involving Optic Radiation. Neurosurgery (2011) 69(5):1070–84. doi: 10.1227/NEU.0b013e3182274841 21654536

[14] D'Andrea G Familiari P Di Lauro A Angelini A Sessa G. Safe Resection of Gliomas of the Dominant Angular Gyrus Availing of Preoperative FMRI and Intraoperative DTI: Preliminary Series and Surgical Technique. World Neurosurg (2016) 87:627–39. doi: 10.1016/j.wneu.2015.10.076 26548825

[15] Bauer PR Reitsma JB Houweling BM Ferrier CH Ramsey NF. Can fMRI Safely Replace the Wada Test for Preoperative Assessment of Language Lateralisation? A Meta-Analysis and Systematic Review. J Neurol Neurosurg Psychiatry (2014) 85(5):581–8. doi: 10.1136/jnnp-2013-305659 23986313

[16] Loddenkemper T Morris HH Moddel G. Complications During the Wada Test. Epilepsy Behav E&B (2008) 13(3):551–3. doi: 10.1016/j.yebeh.2008.05.014 18590981

[17] Mikuni N Takayama M Satow T Yamada S Hayashi N Nishida N. Evaluation of Adverse Effects in Intracarotid Propofol Injection for Wada Test. NEUROLOGY (2005) 65(11):1813–6. doi: 10.1212/01.wnl.0000176988.87336.ff 16148262

[18] Delgado-Fernandez J Garcia-Pallero MA Manzanares-Soler R Martin-Plasencia P Blasco G Frade-Porto N. Language Hemispheric Dominance Analyzed With Magnetic Resonance DTI: Correlation With the Wada Test. J Neurosurg (2020) 134(6):1703–10. doi: 10.3171/2020.4.JNS20456 32707542

[19] Ellmore TM Beauchamp MS Breier JI Slater JD Kalamangalam GP O'Neill TJ. Temporal Lobe White Matter Asymmetry and Language Laterality in Epilepsy Patients. Neuroimage (2010) 49(3):2033–44. doi: 10.1016/j.neuroimage.2009.10.055 PMC2818356 19874899

[20] Matsumoto R Okada T Mikuni N Mitsueda-Ono T Taki J Sawamoto N. Hemispheric Asymmetry of the Arcuate Fasciculus: A Preliminary Diffusion Tensor Tractography Study in Patients With Unilateral Language Dominance Defined by Wada Test. J Neurol (2008) 255(11):1703–11. doi: 10.1007/s00415-008-0005-9 18821045

[21] Tiwari VN Jeong JW Asano E Rothermel R Juhasz C Chugani HT. A Sensitive Diffusion Tensor Imaging Quantification Method to Detect Language Laterality in Children: Correlation With the Wada Test. J Child Neurol (2011) 26(12):1516–21. doi: 10.1177/0883073811409225 PMC3673532 21652590

[22] Zhao Y Chen X Wang F Sun G Wang Y Song Z. Integration of Diffusion Tensor-Based Arcuate Fasciculus Fibre Navigation and Intraoperative MRI Into Glioma Surgery. J Clin Neurosci (2012) 19(2):255–61. doi: 10.1016/j.jocn.2011.03.041 22273119

[23] Li FY Liu HY Zhang J Sun ZH Zhang JS Sun GC. Identification of Risk Factors for Poor Language Outcome in Surgical Resection of Glioma Involving the Arcuate Fasciculus: An Observational Study. Neural Regener Res (2021) 16(2):333–7. doi: 10.4103/1673-5374.290901 PMC7896210 32859793

[24] Oldfield RC. The Assessment and Analysis of Handedness: The Edinburgh Inventory. Neuropsychologia (1971) 9(1):97–113. doi: 10.1016/0028-3932(71)90067-4 5146491

[25] Andrew K. Western Aphasia Battery. 1st Ed. San Antonio, TX: The Psychological Corporation (1982).

[26] Fujii M Miyachi S Matsubara N Kinkori T Takebayashi S Izumi T. Selective Propofol Injection Into the M1 Segment of the Middle Cerebral Artery (MCA Wada Test) Reduces Adverse Effects and Enhances the Reliability of the Wada Test for Determining Speech Dominance. World Neurosurg (2011) 75(3-4):503–8. doi: 10.1016/j.wneu.2010.10.048 21600504

[27] Mikuni N Yokoyama Y Matsumoto A Kikuchi T Yamada S Hashimoto N. Intravenous Methylprednisolone Reduces the Risk of Propofol-Induced Adverse Effects During Wada Testing. Neurol Med Chir (Tokyo) (2010) 50(8):622–6. doi: 10.2176/nmc.50.622 20805642

[28] Catani M Howard RJ Pajevic S Jones DK. Virtual in Vivo Interactive Dissection of White Matter Fasciculi in the Human Brain. NeuroImage (2002) 17(1):77–94. doi: 10.1006/nimg.2002.1136 12482069

[29] Fernandez-Miranda JC Wang Y Pathak S Stefaneau L Verstynen T Yeh FC. Asymmetry, Connectivity, and Segmentation of the Arcuate Fascicle in the Human Brain. Brain Struct Funct (2015) 220(3):1665–80. doi: 10.1007/s00429-014-0751-7 24633827

[30] Wada J TR. Intracarotid Injection of Sodium Amytal for the Lateralization of Cerebral Speech Dominance. 1960. J Neurosurg (2007) 106(6):1117–33. doi: 10.3171/jns.2007.106.6.1117 17564192

[31] Krieg SM Sollmann N Hauck T Ille S Foerschler A Meyer B. Functional Language Shift to the Right Hemisphere in Patients With Language-Eloquent Brain Tumors. PloS One (2013) 8(9):e75403. doi: 10.1371/journal.pone.0075403 24069410 PMC3775731

[32] Thiel A Habedank B Herholz K Kessler J Winhuisen L Haupt WF. From the Left to the Right: How the Brain Compensates Progressive Loss of Language Function. Brain Lang (2006) 98(1):57–65. doi: 10.1016/j.bandl.2006.01.007 16519926

[33] Jehna M Becker J Zaar K von Campe G Mahdy Ali K Reishofer G. Symmetry of the Arcuate Fasciculus and Its Impact on Language Performance of Patients With Brain Tumors in the Language-Dominant Hemisphere. J Neurosurg (2017) 127(6):1407–16. doi: 10.3171/2016.9.JNS161281 28128689

[34] Henderson F Abdullah KG Verma R Brem S. Tractography and the Connectome in Neurosurgical Treatment of Gliomas: The Premise, the Progress, and the Potential. Neurosurg Focus. (2020) 48(2):E6. doi: 10.3171/2019.11.FOCUS19785 PMC7831974 32006950

[35] Bello L Gambini A Castellano A Carrabba G Acerbi F Fava E. Motor and Language DTI Fiber Tracking Combined With Intraoperative Subcortical Mapping for Surgical Removal of Gliomas. Neuroimage (2008) 39(1):369–82. doi: 10.1016/j.neuroimage.2007.08.031 17911032

[36] Abou-Khalil B. An Update on Determination of Language Dominance in Screening for Epilepsy Surgery: The Wada Test and Newer Noninvasive Alternatives. Epilepsia (2007) 48(3):442–55. doi: 10.1111/j.1528-1167.2007.01012.x 17319925

[37] Meinhold T Hofer W Pieper T Kudernatsch M Staudt M. Presurgical Language fMRI in Children, Adolescents and Young Adults : A Validation Study. Clin Neuroradiol (2020) 30(4):691–704. doi: 10.1007/s00062-019-00852-7 31960077

[38] Dym RJ Burns J Freeman K Lipton ML. Is Functional MR Imaging Assessment of Hemispheric Language Dominance as Good as the Wada Test?: A Meta-Analysis. Radiology (2011) 261(2):446–55. doi: 10.1148/radiol.11101344 21803921

[39] Woermann FG Jokeit H Luerding R Freitag H Schulz R Guertler S. Language Lateralization by Wada Test and fMRI in 100 Patients With Epilepsy. Neurology (2003) 61(5):699–701. doi: 10.1212/01.WNL.0000078815.03224.57 12963768

[40] Orringer DA Vago DR Golby AJ. Clinical Applications and Future Directions of Functional MRI. Semin Neurol (2012) 32(4):466–75. doi: 10.1055/s-0032-1331816 PMC3787513 23361489

[41] Ulmer JL Hacein-Bey L Mathews VP Mueller WM DeYoe EA Prost RW. Lesion-Induced Pseudo-Dominance at Functional Magnetic Resonance Imaging: Implications for Preoperative Assessments. Neurosurgery (2004) 55(3):569–79. doi: 10.1227/01.NEU.0000134384.94749.B2 15335424

[42] Wellmer J Weber B Urbach H Reul J Fernandez G Elger CE. Cerebral Lesions can Impair fMRI-Based Language Lateralization. Epilepsia (2009) 50(10):2213–24. doi: 10.1111/j.1528-1167.2009.02102.x 19453706

[43] Zacà D Agarwal S Gujar SK Sair HI Pillai JJ. Special Considerations/Technical Limitations of Blood-Oxygen-Level-Dependent Functional Magnetic Resonance Imaging. Neuroimaging Clinics North Am (2014) 24(4):705–15. doi: 10.1016/j.nic.2014.07.006 25441509

[44] Raffa G Quattropani MC Germano A. When Imaging Meets Neurophysiology: The Value of Navigated Transcranial Magnetic Stimulation for Preoperative Neurophysiological Mapping Prior to Brain Tumor Surgery. Neurosurg Focus (2019) 47(6):E10. doi: 10.3171/2019.9.FOCUS19640 31786549

[45] Raffa G Conti A Scibilia A Sindorio C Quattropani MC Visocchi M. Functional Reconstruction of Motor and Language Pathways Based on Navigated Transcranial Magnetic Stimulation and DTI Fiber Tracking for the Preoperative Planning of Low Grade Glioma Surgery: A New Tool for Preservation and Restoration of Eloquent Networks. Acta Neurochir Suppl (2017) 124:251–61. doi: 10.1007/978-3-319-39546-3_37 28120081

[46] Thiebaut de Schotten M Ffytche DH Bizzi A Dell'Acqua F Allin M Walshe M. Atlasing Location, Asymmetry and Inter-Subject Variability of White Matter Tracts in the Human Brain With MR Diffusion Tractography. Neuroimage (2011) 54(1):49–59. doi: 10.1016/j.neuroimage.2010.07.055 20682348

[47] Catani M Allin MP Husain M Pugliese L Mesulam MM Murray RM. Symmetries in Human Brain Language Pathways Correlate With Verbal Recall. Proc Natl Acad Sci U S A (2007) 104(43):17163–8. doi: 10.1073/pnas.0702116104 PMC2040413 17939998

[48] Duffau H Moritz-Gasser S Mandonnet E. A Re-Examination of Neural Basis of Language Processing: Proposal of a Dynamic Hodotopical Model From Data Provided by Brain Stimulation Mapping During Picture Naming. Brain Lang (2014) 131:1–10. doi: 10.1016/j.bandl.2013.05.011 23866901

[49] Silva G Citterio A. Hemispheric Asymmetries in Dorsal Language Pathway White-Matter Tracts: A Magnetic Resonance Imaging Tractography and Functional Magnetic Resonance Imaging Study. Neuroradiol J (2017) 30(5):470–6. doi: 10.1177/1971400917720829 PMC5602342 28699372

[50] Vassal F Schneider F Boutet C Jean B Sontheimer A Lemaire JJ. Combined DTI Tractography and Functional MRI Study of the Language Connectome in Healthy Volunteers: Extensive Mapping of White Matter Fascicles and Cortical Activations. PloS One (2016) 11(3):e0152614. doi: 10.1371/journal.pone.0152614 27029050 PMC4814138

[51] Campbell JS Pike GB. Potential and Limitations of Diffusion MRI Tractography for the Study of Language. Brain Lang (2014) 131:65–73. doi: 10.1016/j.bandl.2013.06.007 23910928

[52] Bauer MH Kuhnt D Barbieri S Klein J Becker A Freisleben B. Reconstruction of White Matter Tracts *via* Repeated Deterministic Streamline Tracking–Initial Experience. PloS One (2013) 8(5):e63082. doi: 10.1371/journal.pone.0063082 23671656 PMC3646033

[53] Richter M Zolal A Ganslandt O Buchfelder M Nimsky C Merhof D. Evaluation of Diffusion-Tensor Imaging-Based Global Search and Tractography for Tumor Surgery Close to the Language System. PloS One (2013) 8(1):e50132. doi: 10.1371/journal.pone.0050132 23308093 PMC3538752

[54] Yeh FC Verstynen TD Wang Y Fernandez-Miranda JC Tseng WY. Deterministic Diffusion Fiber Tracking Improved by Quantitative Anisotropy. PloS One (2013) 8(11):e80713. doi: 10.1371/journal.pone.0080713 24348913 PMC3858183

[55] Farquharson S Tournier JD Calamante F Fabinyi G Schneider-Kolsky M Jackson GD. White Matter Fiber Tractography: Why We Need to Move Beyond DTI. J Neurosurg (2013) 118(6):1367–77. doi: 10.3171/2013.2.JNS121294 23540269

[56] Sydnor VJ Rivas-Grajales AM Lyall AE Zhang F Bouix S Karmacharya S. A Comparison of Three Fiber Tract Delineation Methods and Their Impact on White Matter Analysis. Neuroimage (2018) 178:318–31. doi: 10.1016/j.neuroimage.2018.05.044 PMC6481642 29787865

[57] Hill VB Cankurtaran CZ Liu BP Hijaz TA Naidich M Nemeth AJ. A Practical Review of Functional MRI Anatomy of the Language and Motor Systems. AJNR Am J Neuroradiol (2019) 40(7):1084–90. doi: 10.3174/ajnr.A6089 PMC6754743 31196862

